# Brief Overview of Ice Nucleation

**DOI:** 10.3390/molecules26020392

**Published:** 2021-01-13

**Authors:** Nobuo Maeda

**Affiliations:** Department of Civil & Environmental Engineering, School of Mining and Petroleum Engineering, University of Alberta, 7-207 Donadeo ICE, 9211-116 Street NW, Edmonton, AB T6G1H9, Canada; nobuo@ualberta.ca; Tel.: +1-780-492-6524

**Keywords:** nucleation, ice, heterogeneous nucleation, classical nucleation theory, organic ice nucleator, memory effect, surface nucleation, immersion nucleation, contact nucleation, multi-step nucle-ation

## Abstract

The nucleation of ice is vital in cloud physics and impacts on a broad range of matters from the cryopreservation of food, tissues, organs, and stem cells to the prevention of icing on aircraft wings, bridge cables, wind turbines, and other structures. Ice nucleation thus has broad implications in medicine, food engineering, mineralogy, biology, and other fields. Nowadays, the growing threat of global warming has led to intense research activities on the feasibility of artificially modifying clouds to shift the Earth’s radiation balance. For these reasons, nucleation of ice has been extensively studied over many decades and rightfully so. It is thus not quite possible to cover the whole subject of ice nucleation in a single review. Rather, this feature article provides a brief overview of ice nucleation that focuses on several major outstanding fundamental issues. The author’s wish is to aid early researchers in ice nucleation and those who wish to get into the field of ice nucleation from other disciplines by concisely summarizing the outstanding issues in this important field. Two unresolved challenges stood out from the review, namely the lack of a molecular-level picture of ice nucleation at an interface and the limitations of classical nucleation theory.

## 1. Introduction

The nucleation of ice is vital in cloud physics and impacts on a broad range of matters, from the cryopreservation of food, tissues, organs, and stem cells [1,2,3,4,5,6,7,8,9,10,11,12,13,14,15] to the prevention of icing on aircraft wings, bridge cables, wind turbines, and other structures [16,17,18,19,20,21,22]. Ice nucleation thus has broad implications in medicine, food engineering, mineralogy, biology, and other fields. Nowadays, the growing threat of global warming led to intense research activities on feasibility of artificially modifying clouds to shift the Earth’s radiation balance, which has launched a sort of renaissance in cloud physics [23,24,25,26,27,28]. In short, formation of icy clouds at appropriate altitudes that scatter incoming sunlight (the albedo) might offset global warming associated with the increased concentrations of greenhouse gases. For these reasons, the nucleation of ice has been extensively studied over many decades, and rightfully so.

Because of the sheer number of research activities in the field, it is not quite possible to cover the whole subject of ice nucleation in a single review. Readers who are interested in a role that ice nucleation plays in the specific examples listed above are therefore referred to the recent review articles in the respective sub-fields. This feature article does not attempt to cover such a broad range of topics. Rather, it only reviews our understanding of ice nucleation as a physical process and focuses on several major outstanding fundamental issues that stand in the way of our understanding. The author’s wish is to aid early researchers in the field of ice nucleation and those who wish to get into the field of ice nucleation from other disciplines by concisely summarizing the outstanding issues in the field. To this end, we start from briefly reviewing the essence of nucleation and classical nucleation theory, and then look into the specific issues that pose challenges to our physical understanding of the subject. Similar questions have been asked from time to time in the past [29,30], and given the advances made in the field since the time of these reports, it appears pertinent to re-ask these outstanding questions. 

## 2. Nucleation and Classical Nucleation Theory

It is commonly observed that condensation of a vapor into a liquid does not occur at the boiling point of the substance or freezing of a liquid into a solid does not occur at the melting point of the substance, but instead requires an excess subcooling or supercooling. A similar observation may be made for boiling of a liquid that requires superheating. This ability of a substance to subcool, supercool, or superheat beyond a thermodynamic phase boundary in the process of a first order phase transition is the subject of nucleation. 

The most common theoretical framework that deals with the subject of nucleation is classical nucleation theory [31]. Unfortunately, the phrase “classical nucleation theory” has not been very well defined in the literature, however, which appears to cause confusions from time to time. The definition of a phase requires spatial uniformity of physical properties within the phase. Classical nucleation theory in the broadest sense refers to a theoretical framework that allows *temporary* formations of spatially non-uniform patches of a secondary phase within the original parent phase during a transition from the original phase to the secondary phase [31]. In a narrower sense, the same phrase may refer to a particular aspect of said broadest definition. 

It turned out that many first-order phase transitions are activation processes that require surmounting of an activation barrier (melting of most solids are exceptions [32,33]). Freezing of liquid water to form ice is an example of such a phase transition. Because of this activation barrier, some first-order phase transitions do not occur at a well-defined pressure–temperature condition but over a range of such conditions. Microscopically, this process of surmounting of an activation barrier, i.e., nucleation, is a process of random generation of small formations of the new, thermodynamically stable phase (nucleus) that have the ability for irreversible overgrowth to macroscopic sizes [31]. 

The probability of such occurrence depends on the *driving force* for nucleation, which is the *chemical potential differential* between the thermodynamically stable phase and the metastable parent phase [31]. Thus, as would be expected, nucleation can only occur below the melting point for freezing, below the boiling point for condensation or above the boiling point for boiling. The chemical potential differential progressively widens as the system subcools, supercools, or superheats more, and consequently the driving force for nucleation progressively increases with the system subcooling, subcooling or superheating. 

An important contribution of classical nucleation theory is that it can quantitatively relate the *nucleation rate* (nucleation frequency) to the *nucleation work* that is required to surmount an activation barrier. The nucleation rate can generally be expressed in the form of a product of a kinetic factor, which accounts for the frequency with which the system “attempts” to realize a nucleation event, and a thermodynamic factor, which accounts for the probability that each such “attempt” results in actual surmounting of said activation barrier [31]. The kinetic factor centers on the attachment frequency of monomer molecules to an incipient cluster, which reflects the mobility of the monomer molecules in a supercooled condition. The thermodynamic parameter expresses the probability of finding a system in a state that has a sufficient amount of free energy to surmount the activation barrier. These considerations lead to an equation of the Arrhenius form which results from the Boltzmann distribution of classical statistical physics.

*J* = *AN*_0_ exp (−Δ*g*/*kT*) (1)

where *J* is the nucleation rate, *A* is a kinetic constant that includes the attachment frequency of monomer molecules, *N*_0_ is the concentration of potential nucleation sites, Δ*g* is the height of the activation barrier (or the nucleation work that is required to surmount said activation barrier), *k* is the Boltzmann constant, and *T* is the absolute temperature. *J* is often expressed in the form of (*J*/*N*_0_) with an assumption that *N*_0_ is proportional to the system volume for homogeneous nucleation and proportional to the surface area for heterogeneous nucleation. It is further assumed that Δ*g* is the same over all the *N*_0_ sites. 

The physical source of the activation barrier is the interfacial free energy between the thermodynamically stable phase and the metastable parent phase [31]. Before the thermodynamically stable phase eventually consumes the entire metastable parent phase, the system necessarily exposes an interface between the two phases. Creation of such an interface costs free energy which is given by the product of the interfacial area and the specific interfacial free energy between a thermodynamically stable phase and a metastable parent phase [31]. In this feature article, we use the term “specific interfacial free energy” to refer to the interfacial free energy of a unit area of an interface between two well-defined media that has a unique value under a given temperature and pressure [34]. Likewise, we use the term “specific surface energy” for the surface energy of a unit area of a well-defined surface in vacuum that has a unique value under a given temperature and pressure. In practice, the vacuum may be substituted by the vapor of the solid in question. 

**Figure 1:**
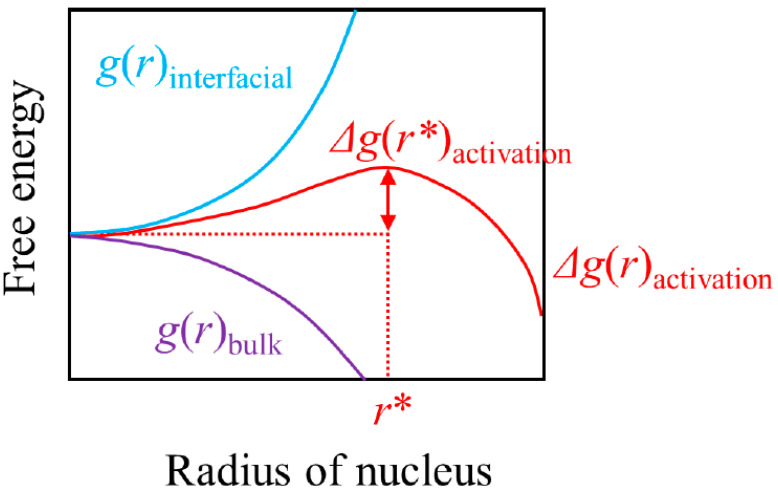
Schematic illustration of the activation barrier. The figure is shown for a constant temperature/pressure for which the prevailing phase is metastable.

We show a schematic illustration of the activation barrier in Figure 1 for a rather ideal case of a spherical nucleus during homogeneous nucleation. Here, *r* is the radius of said spherical nucleus, *g*(*r*)_bulk_ is the free energy of forming a new phase that arises from the bulk contribution of bringing atoms or molecules together, *g*(*r*)_interfacial_ is the interfacial free energy between the new phase and the old phase and Δ*g*(*r*)_activation_ is the sum of the two.

Δ*g*(*r*)_activation_*= g*(*r*)_bulk_ + *g*(*r*)_interfacial_ (2)

We note that all the three curves of *g*(*r*)_bulk_, *g*(*r*)_interfacial_ and Δ*g*(*r*)_activation_ are functions of temperature and pressure, and that Figure 1 is shown *for a constant temperature and pressure for which the prevailing phase is metastable*. The figure would look very different depending on the temperature/pressure in question [34]. 

At a temperature/pressure condition that is moderately subcooled, supercooled or superheated, and the prevailing phase is thermodynamically metastable (a situation that corresponds to the description in Figure 1), formation of a thermodynamically stable phase would lower *g*(*r*)_bulk_ and the *g*(*r*)_bulk_ term will fall progressively as the thermodynamically stable phase grows. Thus, the *g*(*r*)_bulk_ term will monotonically fall with *r* and the size of the fall is proportional to the volume of the nucleus, i.e., proportional to the cube of *r*. In contrast, the *g*(*r*)_interfacial_ term is always positive in the relevant range of temperatures/pressures and is proportional to the interfacial area between the new and the old phases, i.e., proportional to the square of *r*. We note at this stage that the specific interfacial free energy gradually falls with increasing temperature and eventually disappears at the critical point. Since, for the temperature/pressure we are considering now, *g*(*r*)_bulk_ is proportional to *r*^3^ and *g*(*r*)_interfacial_ is proportional to *r*^2^, and Δ*g*(*r*)_activation_ *= g*(*r*)_bulk_ + *g*(*r*)_interfacial_, there will be a maximum in Δ*g*(*r*)_activation_ somewhere *r* > 0. The size of the nucleus that corresponds to this maximum, r*, is called the *critical nucleus size*, and the thermodynamically stable phase can continue growing once it somehow finds a way to grow larger than this r*. The corresponding value in Δ*g*(*r*)_activation_, Δ*g*(r*)_activation_, is called the activation barrier and is numerically identical to the nucleation work that is required to surmount said activation barrier. 

At a temperature for which the driving force is very large, the *g*(*r*)_bulk_ term falls off almost vertically at a very small *r* value toward negative infinity. Then, the positive *g*(*r*)_interfacial_ term would not be sufficient in magnitude to render Δ*g*(*r*)_activation_ positive for any *r* that is larger than the size of an atom or a molecule. Consequently, Δ*g*(*r*)_activation_ also falls off at *r* ≈ 0 toward negative infinity. Then, the maximum in Δ*g*(*r*)_activation_, Δ*g*(r*)_activation_, will be realized at *r* ≈ 0 and the height of the activation barrier will be practically zero. Thus, no activation barrier would be required to overcome in such an extreme case and the phase transition will proceed with certainty. In the case of freezing of a liquid, however, such a rapid quenching of a liquid would “petrify” the motion of the molecules involved and the phase transition may not occur immediately, notwithstanding the total absence of an activation barrier (this is to say that the kinetic factor in Equation (1) becomes very small) [35]. This factor, *viscous slowdown*, is of a different nature from the factor that arises from an activation process. 

In contrast, at a temperature for which the prevailing phase is thermodynamically stable, both *g*(*r*)_bulk_ and *g*(*r*)_interfacial_ are positive for all *r* and both monotonically increase with *r* toward (positive) infinity. Therefore, Δ*g*(*r*)_activation_ is also positive for all *r* and monotonically increases with *r* toward infinity. Consequently, the minimum of Δ*g*(*r*)_activation_ can only be realized at *r* = 0 and the maximum of Δ*g*(*r*)_activation_, Δ*g*(r*)_activation_, diverges to infinity. No phase transition can possibly proceed under such a temperature. 

A salient point here is that a cluster that is smaller than the critical size at a moderately subcooled, supercooled or superheated temperature actually has a higher free energy than that of an ensemble of monomers that comprise the cluster because of the *g*(*r*)_interfacial_ term. In other words, such clusters cannot exist at all in a system that is in thermodynamic equilibrium. However, they may exist temporarily. Such temporary formations of thermodynamically unstable clusters are at the heart of nucleation theory and this is possible because of the uncertainty principle of quantum mechanics [34]. Simply put, the lifetime of an unrealistically high energy state is very short, but not zero, and the lifetime progressively increases as the energy level becomes lower and more realistic. Then, the probability that a monomer attaches to a sub-critically sized cluster, which is thermodynamically unstable and hence can only exist briefly, increases with the lifetime of the cluster. Because of this non-zero probability of the system to “get around” or “tunnel through” the activation barrier, nucleation becomes an intrinsically stochastic event under a modest metastability (i.e., before the driving force becomes so large that the height of the activation barrier falls all the way to zero) [34]. It is therefore experimentally impossible to “repeat” or “reproduce” a nucleation event, in a deterministic sense, because the nature of the phenomenon is probabilistic. Instead, a probabilistic approach based on a large number of measurements of metastable systems is generally required (it remains open as to how best to analyze such statistical data [34,36]). Theoretically, this intrinsically probabilistic nature of nucleation is incorporated in the form of probability distributions of the Boltzmann form of classical statistical physics [37]. 

For the simplest case of the condensation of vapor into a liquid of a single-component system (i.e., in the absence of any other entity which corresponds to homogeneous nucleation), the Kelvin equation relates the chemical potential increment due to the supersaturation of the vapor to the chemical potential increment due to the positive curvature of the interface between the condensing phase and the metastable vapor phase (i.e., the positive Laplace pressure) [38,39].

*kT* ln(*P*/*P*_0_) = γ*v*_m_/*r*_K_ (3)

where *k* is the Boltzmann constant, *T* is the absolute temperature, *P* is the actual vapor pressure, *P*_0_ is the saturation vapor pressure, γ is the specific interfacial free energy, *v*_m_ is the molecular volume and *r*_K_ is the Kelvin radius. For a spherical nucleus, *r* = 2*r*_K_ where *r* is the radius of the nucleus, and *r* is defined positive for a convex interface (like a droplet) and negative for a concave interface (like a meniscus). The Kelvin equation can thus provide the critical nucleus size of a condensing droplet in a supersaturated vapor when the free energy increment becomes the maximum, which corresponds to the apex of the activation barrier we discussed with Figure 1 earlier. As expected, the Kelvin radius progressively becomes smaller as the supersaturation of the vapor mounts, i.e., the vapor is only required to produce a progressively smaller nucleus as the supersaturation mounts before said nucleus can grow on its own to a macroscopically large mass of liquid. Perhaps, also as expected, a greater supersaturation is required to homogeneously nucleate a liquid which has a larger specific surface free energy that would raise the free energy increment. 

The situation is more complex for a liquid-to-solid phase transition below the melting point because a crystal is generally anisotropic. The specific interfacial free energy between a thermodynamically stable crystalline phase and its metastable parent liquid phase is not only a function of temperature and pressure [38]; a crystal generally has multiple crystallographic *facets* and each facet has its own unique specific interfacial free energy that varies with the temperature and pressure. 

**Figure 2:**
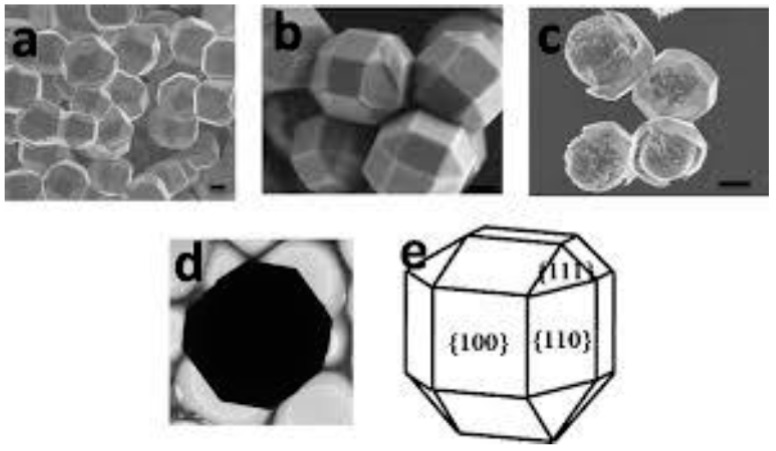
The Wulff theorem. The size of the area that a facet exposes to its surrounding medium is inversely proportional to the specific interfacial free energy value between that facet and the surrounding medium. Image reproduced from [41], with permission from Royal Society of Chemistry.

For homogeneous nucleation, that is, nucleation in the absence of a third party, the relative size of the specific interfacial free energy values among different facets between a crystalline phase and its melt can be inferred from the *shape* of a slowly growing single crystal that is in quasi-equilibrium with its surrounding melt, in accordance with the Wulff theorem [40]. In short, the lowest free energy of a system can be achieved, in a quasi-equilibrium, when the sum of the interfacial free energy values of all the facets of a growing crystal becomes the minimum. Thus, the size of the area of a crystallographic facet which a slowly growing single crystal exposes to its surrounding medium is inversely proportional to the specific interfacial free energy value between that facet and the surrounding medium (Figure 2). Then, *a crystallographic facet that has the lowest specific interfacial free energy with its surrounding medium will become the preferred facet that nucleates.* Thus, the relevant interfacial free energy in the nucleation work is that of the crystallographic facet of the lowest specific interfacial free energy value. Though conceptually clear, it is very difficult in practice to measure the specific interfacial energy between a given crystallographic facet and its melt. 

For heterogeneous nucleation, that is, nucleation in the presence of a third party, it has not been feasible to measure the specific interfacial energy between a given crystallographic facet of a nucleating single crystal and a surface of a foreign solid material of interest. Still, the same conceptual framework is assumed to apply—a crystallographic facet of the nucleating crystal that has the lowest specific interfacial free energy value with the surface of the foreign material of interest becomes the nucleation center from which the new crystalline phase grows. Pragmatically, the situation is not as bad as it might appear. Since the crystallographic facet of the lowest specific interfacial free energy value is expected to expose the largest interfacial area, its relative weight to the average specific interfacial free energy should be large. Then, an experimentally measured specific interfacial free energy value, if it can be considered to represent a weighted average over all the facets, could be reasonably close to that of the facet of the lowest specific interfacial free energy. Measurements of such an average specific interfacial free energy is not a trivial matter either, nevertheless, should be easier than measurements of the specific interfacial free energy of individual crystallographic facets. 

It is important to re-iterate that a simple schematic illustration shown in Figure 1 is for homogeneous nucleation that can only occur in very limited circumstances. Another major contribution of classical nucleation theory is that it can quantitatively relate the height of the homogeneous activation barrier to the height of the heterogeneous activation barrier. In short, classical nucleation theory expresses the extent of “catalyzing effect” of heterogeneous nucleation in terms of the size of the activation barrier. For the simplest case of a spherical-cap-shaped nucleus, the free energy of forming a spherical-cap-shaped nucleus consisting of *n* molecules on a foreign solid substrate (heterogeneous nucleation) is related to that of a spherical nucleus of the same number of *n* molecules in the homogeneous nucleation in terms of the height of the activation barrier [31,34];

Δ*g*_activation, heterogeneous_/Δ*g*_activation, homogeneous_ = [(1/4)(2 + cosθ)(1 − cosθ)^2^]^1/3^ (4)

where θ is the contact angle of a spherical cap–shaped nucleus that forms on the substrate in the metastable parent phase. 

Equation (4) shows that the activation barrier of heterogeneous nucleation steadily falls as the affinity or “wettability” of the foreign substrate to the nucleating phase improves, from a limiting case of the same size as the homogeneous nucleation to the other limiting case of zero. Thus, presence of a foreign solid particle would substantially lower the activation barrier, at times almost to zero. Even a solid particle that has a very poor affinity to the nucleating phase would still lower the activation barrier from that of homogeneous nucleation, with the sole exception occurring when the “contact angle” is 180° (i.e., when the substrate has no affinity at all; note that even in this limiting case the activation barrier is not any higher than that of the homogeneous nucleation). The exponential dependence of the nucleation rate to the activation barrier in Equation (1) shows that homogeneous nucleation is extremely rare in reality for these reasons. To this end, we will concentrate ourselves mainly to heterogeneous nucleation of ice in the remainder of this feature article. 

To summarize, classical nucleation theory has been built on a conceptually simple probabilistic framework of the Boltzmann distribution. There are three major limitations in classical nucleation theory, however. First, experimental detections of nucleation events implicitly assume that formation of a single critically-sized nucleus somewhere in a given system results in a macroscopic phase transition of the whole system. Even if multiple critically sized nuclei were to form simultaneously, the thermodynamically stable phase that grows from each critically-sized nuclei would merge and result in only one experimentally detectable phase transition. It is not experimentally feasible to individually detect such multiple simultaneous nucleation events unless such nucleation events are separated by macroscopic distances. Only counting one of such multiple nucleation events would lead to effective undercounting of nucleation events and hence result in experimentally determined nucleation rates to be underestimated. The “real” nucleation rate, if each such individual nucleation event were separately detectable, would have been higher. 

Second, the presence of cooperative phenomena that differ from the totally random Arrhenius behavior would limit the applicability of classical nucleation theory to such a system [42]. Classical nucleation theory is built on a random chance of surmounting of an activation barrier. Equation (1) shows that the chance of nucleation (*probability density* of nucleation), *J*, is a simple product of the frequency with which a given system attempts to surmount an activation barrier (the kinetic factor) and the probability that each such attempt actually resulting in surmounting of the activation barrier (the thermodynamic factor). No provisions have been made to incorporate any cooperative phenomena in the theoretical framework of classical nucleation theory. 

Third, classical nucleation theory implicitly assumes that the nucleating phase is the thermodynamically stable phase. A different approach would be required when a phase transition were to proceed via a transient, thermodynamically metastable third phase. Nucleation most likely proceeds from the nucleus with a crystallographic facet that has the smallest specific interfacial free energy value with the surrounding medium, which is not necessarily a facet of the thermodynamically stable crystal. We will see such an example for two ice phases later. In addition, as we will also see later, ice nucleation from water vapor, for which ice is the thermodynamically stable phase as opposed to liquid water, could in some cases proceed in two steps of (1) condensation of (thermodynamically metastable) water vapor followed by (2) the freezing of the condensed liquid water. Such multi-step nucleation processes are a norm for the nucleation of clathrate hydrates [34], and so appears the case for ice. Experimentally, only the *effective* nucleation rate for a whole nucleation event (i.e., only the end result of a given phase transition) can often be detected, regardless of how many kinetic steps are involved in the nucleation process. In other words, an experimentally determined nucleation rate corresponds to the *nucleation probability density* of all the kinetic hurdles being surmounted *in series* (i.e., in a single continuous path). Thus, for a theory to match an experimentally determined nucleation rate, each sub-component of the multi-step nucleation process needs to be incorporated. If one knows *a priori* (1) how many kinetic steps are present in a given nucleation process and (2) the kinetic factor and the size of the activation barrier of each such kinetic step, then the (experimentally measurable) overall nucleation rate could be theoretically given by the product of all the kinetic factors and the thermodynamic factors involved. 

## 3. Nucleation of Ice from Liquid Water

A solid crystal can nucleate from its liquid melt or from its vapor and so is the case for ice. Homogeneous nucleation of ice in liquid water can only occur in very limited circumstances and consequently most ice nucleation is through heterogeneous nucleation. Perhaps surprisingly, neither a comprehensive theory nor a standard measurement technique exists for heterogeneous nucleation of ice despite the intense research activities that have been ongoing [30]. For example, a broad range of substances have been found to facilitate heterogeneous nucleation of ice, but it remains elusive to understand (1) why these substances facilitate heterogeneous nucleation of ice, (2) what the most important properties of an effective heterogeneous nucleator are, or (3) how to design an effective heterogeneous nucleator. A reliable standard heterogeneous nucleator would provide a great help in standardizing the baseline from which all the other ice nucleation can be measured and compared. Researchers tried several materials in search of such a standard nucleator, however, it proved challenging because sample variability and heterogeneity (purity, grain size, surface defects, etc.) as well as sample aging caused the “baseline” to shift [43]. 

It has been well known that the freezing point of water can be lowered by an application of hydrostatic pressures due to the negative slope of the ice–liquid water phase boundary of H_2_O. It turned out that the temperature at which the nucleation probability becomes high can also be lowered by an application of hydrostatic pressures [42]. The deep supercooling of water can therefore be further deepened by increasing pressures [44], from approximately 230 K under the atmospheric pressure to approximately 180 K under 0.2 GPa [42]. Such deep supercoolings observed for liquid water are in a sense surprising given that liquid water is already highly structured. Liquid water at ambient temperature can be viewed as an extensively connected network of hydrogen bonds and its connectivity is well above the percolation threshold [45]. A molecular-dynamics simulation study found that defects in the extensive association of the hydrogen bond network provided energetically inexpensive pathways between different tetrahedral local arrangements and improved the molecular mobility in the otherwise highly structured liquid water [45]. This picture is conceptually akin to proton hopping—highly structured liquid water can flow as long as the timescales of the tetrahedral local rearrangements due to the defects in the hydrogen bonds are faster than the timescales of the molecular displacements of the flow. The coordination number of H_2_O only changes from about 4.0 to 4.4 when ice melts and the hydrogen bonding of liquid water only gradually breaks with warming above 273.15 K [35,44]. If freezing of water is a transition from a highly-structured phase to another highly-structured phase, and only marginally changes the coordination number of water molecules, why should the process require surmounting of a large activation barrier? In addition, it was found that the hydrogen-bonding network became progressively more cooperative with cooling [46,47], which is expected to render deep supercooling even more difficult for supercooled water than for other supercooled liquids. On the other hand, X-ray scattering data showed that the correlation lengths of density fluctuations in water are virtually independent of temperature while the number of water molecules incorporated into clusters increased as the supercooled water further cooled [48]. 

Even though the main focus of this feature article is on heterogeneous nucleation, we may note that water has a number of anomalous physical properties of its own that become drastically enhanced on deep supercoolings that approach the homogeneous nucleation temperatures of ice. Some thermodynamic response functions appear to diverge at about 228 K [35,49]. A transition between two phases of liquid water, termed a “low density liquid water” and a “high density liquid water”, has been hypothesized to occur below the homogeneous ice nucleation temperature of ice [50,51,52], and an appearance of the low density liquid water appears to precede an incipience of ice nucleus [53]. We note however that such two distinct liquid phases of the same substance have only been discovered for helium and, in the case of helium, the two distinct liquid phases correspond to those of the two isotopes that have totally different physical properties due to the quantum effects. Nevertheless, a recent experimental study using femtosecond X-ray laser pulses detected a presence of metastable bulk liquid water in unconfined micrometer-sized water droplets down to 227 K, at which point ice nucleated promptly [54]. It may sound counter-intuitive, but it turned out that it is more difficult for a computer simulation study to investigate homogeneous nucleation of ice in pure water than heterogeneous nucleation of ice in confined spaces for which the number of possible molecular configurations are limited [55], which obviously has rendered computational investigations of these issues challenging. What further complicates the matter is a possibility that the structure and/or the dynamics of deeply supercooled water may be spatially inhomogeneous for which more than one type of liquid water could coexist at the same temperature [56,57,58]. 

For heterogeneous nucleation, nucleation promoters of ice have been sought after for decades in relation to their applications to cloud seeding. Silver iodide (AgI) has lattice constants that closely match those of ice to within a few percent [59]. Because of this very good lattice matching with ice, AgI has long been believed to be an excellent nucleation promotor of ice that must be ideal for cloud seeding. Perhaps surprisingly, however, the evidence that AgI really enhances rainfall still remains inconclusive despite the decades of commercial cloud seeding with AgI [59]. Ice has been unexpectedly found to grow as discrete hexagonal islands on an AgI substrate, which is against the expectation that an epitaxial growth of a crystal would manifest as a growth of a uniform film on a lattice–matching substrate [59]. This rather apparent contradiction is not unique to AgI. BaF_2_ is not an effective ice nucleator either, despite its good lattice matching to ice [60]. 

It appears that the orientation of the hydrogen bonds (dipoles) of ice is an important factor that influences the heterogeneous nucleation capability of an underlying substrate [61]. It appears that a substrate that orients dipoles at the surface of nucleating ice parallel to one another is a poor nucleation promoter because the orientation reduces the entropy and consequently raises the free energy of any nuclei growing on the substrate [61]. Thus the basal crystallographic faces of AgI or PbI would be poor ice nucleators and their activity is likely confined to the prism faces [61]. A molecular dynamics simulation study found that structurally identical substrates could both inhibit and promote ice formation, depending on the interaction between the substrate surface and H_2_O molecules [62]. A recent study that exchanged cations on molecularly smooth mica surfaces found that ice nucleation temperature progressively became warmer (required smaller supercoolings) with the valency of the exchanged cation, which suggested that the structure of the hydration shell of the cation is a major factor [63]. 

An issue that further complicates the matter in heterogeneous nucleation of ice on a solid surface in liquid water is an impact of surface roughness. Purely geometrical considerations show that surface roughness enhances heterogeneous nucleation potency of a given solid substrate, regardless of the size of its specific surface free energy and regardless of the size of its specific interfacial free energy with the nucleating phase or with the parent metastable phase [64]. Consequently, nanoscale roughness like etch pits on a substrate surface can have a profound effect on the heterogeneous nucleation of ice [59]. 

## 4. Organic Ice Nucleation Promoters

**Figure 3:**
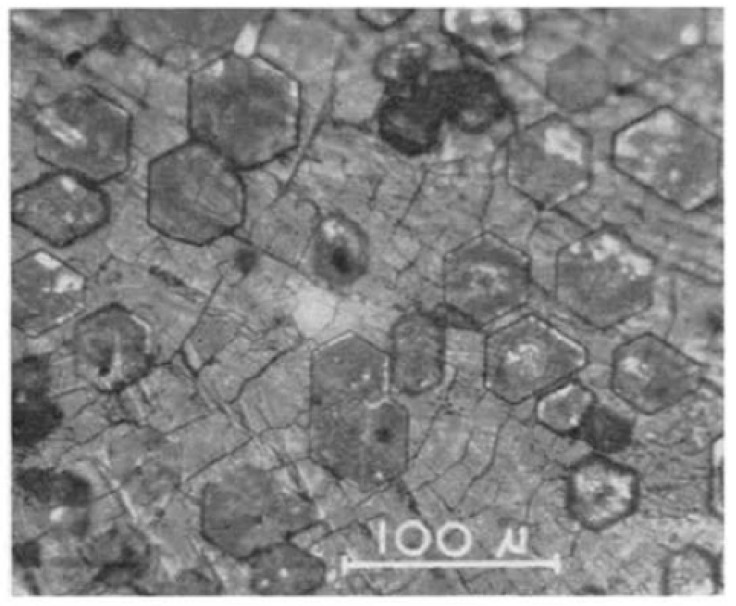
Ice forms on an organic nucleator, like cholesterol shown in the picture, at very small supercoolings in the form of discrete hexagonal crystals, as opposed to continuous two-dimensional films or layers. Image reproduced from [67], with permission from Elsevier.

It was surprising that inorganic crystals that have similar lattice constants to ice, like AgI and BaF_2_, did not promote ice nucleation as much as other substances that had less lattice matching to ice. It is even more surprising that water-insoluble organic compounds with no structural similarities to ice, like steroids and cholesterols [65], and water-soluble macromolecules [66], have been found to effectively nucleate ice. The formation of hexagonal ice crystals on cholesterols that have no lattice matching to ice resulted in surprisingly small supercoolings of down to 1 K (Figure 3) [67]. 

Suspensions of bacteria *Pseudomonas Syringae* have also been found to be an effective nucleator of ice [68,69]. Chemical treatments and physical destruction of the cell destroyed the nucleation potency [68]. A recent study that used surface-sensitive sum frequency generation (SFG) spectroscopy showed that hydrogen bonding at the water-bacteria contact imposed structural ordering on the adjacent water network [69]. It appears that the ice active sites within Pseudomonas Syringae feature unique hydrophilic-hydrophobic patterns to enhance ice nucleation. 

Increasing attention has since been paid to the role of films of high-molecular-weight organic compounds located on water droplets. Films of long-chain alcohols and other organic substances can catalyze ice nucleation in water droplets at a supercooling of only 1 K [70,71,72]. The first stage in the nucleation of ice on organic nucleators, phloroglucinol dehydrate (C_6_H_10_O_5_), was found to be the growth of monolayer patches of ice on the nucleator surface [73]. These are very surprising findings, and more than half a century since the initial report, the underlying mechanisms are still under active investigations [74,75]. We note that these organic compounds generally have lower specific surface energy values than that of water or ice and hence are likely present at the surface of water than in the interior of bulk water, which could give rise to surface nucleation that will be discussed later. 

Another group of unexpected organic ice nucleation promoters is soot [23,25,26,76,77,78]. The surface character of soot particles can have a significant impact on its ice nucleating potency. A molecular dynamics simulation study found that variations in nanostructures alone could account for the spread in the freezing temperatures of ice on soot in experiments, and concluded that a characterization of the nanostructure of soot is needed to predict its ice nucleation efficiency [79]. It appears that chemical groups at the soot surface that can participate in hydrogen bonding with water molecules can increase nucleating potency of ice [80]. Particles synthesized by a method that enhanced the concentration of –OH and carbonyl groups were substantially more efficient as ice nucleator at 253 K than those synthesized by a method that produced particles that were virtually free of –OH and carbonyl groups on the surface. Neutron-scattering studies of water on kerosene soot showed that water was in a liquid-like state on the pore walls of the particles down to 200 K [80]. 

An opposite effect has been observed for the heterogeneous nucleation of ice by poly(vinyl alcohol). The heterogeneous nucleation potency of ice increased with the decreasing density of the –OH groups on poly(vinyl alcohol) [81]. The authors of the study reasoned that the presence of high number densities of the –OH groups constrained the nearby water molecules to re-arrange themselves by their strong mutual interactions and prevented the water molecules from forming an ice structure. Reduction of such constraints by reducing the number density of the –OH groups allowed the water molecules next to the poly(vinyl alcohol) to form ice structure more easily [81]. In contrast, a recent molecular dynamics simulation study found that poly(vinyl alcohol) could increase the nucleation rate by destabilizing water in homogeneous nucleation because the macromolecule is soluble in water and suggested that a potential mechanism in which an organic molecule can increase the nucleation rate of ice may not be limited to heterogeneous nucleation [82]. Clearly, something is amiss though it is not immediately clear what that might be. 

## 5. Nucleation of Ice from Water Vapor

Nucleation of ice in the atmosphere is vital in cloud physics, but nucleation of ice from water vapor is relatively less understood compared to nucleation of ice from liquid water. Supersaturated water vapor at lower altitudes condenses and forms liquid water that becomes rain. Supersaturated water vapor at higher altitudes, for which the thermodynamically stable phase of water is ice (*I*_h_), eventually forms ice that becomes snow or hail. Whether the water vapor in the atmosphere directly nucleates to ice or forms ice through condensation of liquid water is not clear, which in turn casts uncertainty as to the applicability of classical nucleation theory to nucleation of ice from water vapor. In terms of the relative amounts, the great majority of the condensed phase of water in the atmosphere is liquid water and ice is the minority [30]. Though only a minority of the total condensed phases of water in the atmosphere is ice, ice has great impacts on precipitation, cloud electrification, and radiative transfer (the albedo effect) [83,84,85,86,87,88]. 

As we have reviewed above, classical nucleation theory assumes that the critically sized nucleus that overcomes the activation barrier is of the same phase as the thermodynamically stable bulk crystal. It follows that freezing is supposed to be a transition from liquid water to hexagonal ice, *I*_h_, with no intermediate stage. Whether the water vapor in the upper atmosphere directly nucleates to *I*_h_ or first condenses as liquid water and then freezes to *I*_h_ (two-step mechanism) is unclear. A two–step mechanism of (1) condensation of water vapor followed by (2) freezing of the condensed water [89,90] and a three–step mechanism of (1) condensation of water vapor which was followed by (2) freezing of the condensed water which in turn was followed by (3) growth of bulk *I*_h_ from the *I*_h_ nucleus that requires surmounting of an additional activation barrier [91], have been proposed. If the water vapor in the atmosphere does not directly nucleates *I*_h_ but forms *I*_h_ via temporary condensation of metastable liquid water, classical nucleation theory would have trouble in accounting for the experimentally observed nucleation rates or nucleation works. 

**Figure 4:**
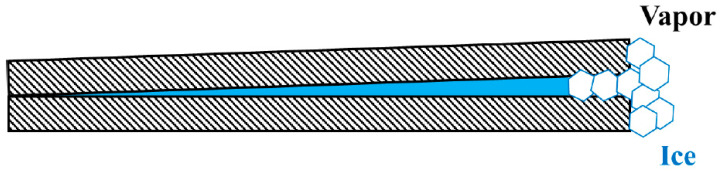
A schematic illustration of condensation of a wetting liquid in a very small wedge when the thermodynamically stable phase of the same macroscopic substance is solid. The very tip of a narrow wedge is effectively one-dimensional and as such condensation can proceed without nucleation (or surmounting of an activation barrier). Presence of a metastable liquid phase could cast doubt as to the applicability of classical nucleation theory to the nucleation of the solid phase.

For condensation of water vapor in the presence of a solid particle, the surface of a solid particle is rarely smooth, and the surface topography becomes an important issue. Here, narrow wedges become preferred condensation sites. The contact of a narrow wedge is effectively one–dimensional and adsorption or condensation in a one-dimensional system does not require any surmounting of an activation barrier [31]. As such, condensation proceeds without nucleation of supersaturation. After water vapor has condensed to such a wedge, however, freezing of the condensed liquid water in the narrow wedge may require surmounting of a large activation barrier of the order of 35 K of supercooling [89,92,93,94]. The reason is that, unlike condensation of “shapeless” liquid, nucleation of a solid phase at the very tip of a narrow wedge that is bounded by two foreign solid walls, like the one schematically depicted in Figure 4, does not occur easily because of the general mismatch between the lattices of the nucleating solid phase and the foreign solid walls and, even in a rare case for which perfect lattice matching is achieved between the two solid phases, the angle of the wedge requires distortion of the lattices that costs extra free energy. Instead, nucleation of the solid phase occurs some distance away from the very tip of the narrow wedge and leaves the trapped liquid next to the very tip unfrozen at small supercoolings [95,96]. In short, the free energy reduction due to the wetting by the liquid is sufficient to offset the free energy cost of keeping the small amount of liquid unfrozen below the melting point when the area-to-volume ratio of the pore geometry is sufficiently large, and this condensation can proceed without any supersaturation of the vapor. 

The consequences of such condensation of the liquid phase in a narrow wedge are two-fold. First, the presence of a metastable liquid phase could cast doubt as to the applicability of classical nucleation theory to the nucleation of the solid phase [89,92]. Second, the condensation would deplete moisture from the surrounding water vapor and by doing so prevent ice from forming elsewhere when the humidity is low [93,94]. When the humidity is high, ice can preferentially form by condensation and freezing on flat, open surfaces [93,94]. 

## 6. System Size Dependence

Nucleation rate of ice depends not only on the temperature and pressure but also on the system size, for example, the size of the droplet. Water droplets of 5-μm radius emulsified in an oil using non-nucleating surfactants appear to supercool to low temperatures similar to those found in cloud chambers [42]. However, perhaps as expected, homogeneous nucleation of *I*_h_ does not occur down to much lower temperatures when the size of the droplet is smaller than the critical nucleus size [97]. As for the relevant scale in question, a recent experimental study that doped liquid water with nanoscale graphene oxide sheets of controlled sizes found that the doped nanoscale graphene oxide sheets only had an impact on the heterogeneous nucleation of ice when their sizes are above a certain threshold value, which the authors reasoned to reflect the size of the critical nucleus for heterogeneous nucleation of ice on graphene oxide of the order of 10 nm [98]. Both theory and experiments show the possibility of an intermediate stage for such a small system [99]. For instance, water clusters of 4000–6000 molecules supercooled down to 200 K and froze to cubic *I*_c_, not hexagonal *I*_h_ [100,101]. Previous studies using different techniques [102,103] and direct detection in the atmosphere [104,105] also showed formation of cubic ice. Like supercooled water, the cubic form of ice is not a thermodynamically stable phase. If supercooled water droplets nucleate as *I*_c_, a cloud that consists of *I*_c_ would equilibrate with a surrounding vapor of a higher relative humidity than a cloud that consists of *I*_h_ would, because the vapor pressure over a metastable phase will always be higher than that over a thermodynamically stable one. Because *I*_h_ has a lower vapor pressure than *I*_c_, ice crystals that have converted from *I*_c_ to *I*_h_ will grow at the expense of the ice crystals that have not. 

Another surprising finding is that, in nano–porous alumina, liquid water supercools to 230 K and then freezes to (metastable) cubic *I*_c_, not to (thermodynamically stable) hexagonal *I*_h_ [106]. *I*_c_ so formed remains metastable during heating, up to 273 K at which point it melts to liquid water [106]. Nanoporous alumina had one of the highest surface to volume ratios of solid walls to date in experimental investigations of nucleation of ice. Such high surface-to-volume ratios are expected to favor heterogeneous nucleation of ice more than ever, and yet liquid water confined inside the nano–porous alumina somehow managed to supercool down to 230 K. We note that, although the pores of the nano–porous alumina were small, they were not too small for a long–range positional order that defines a crystalline phase, i.e., sufficiently large for a metastable *I*_c_ crystalline phase to form [106]. 

**Figure 5:**
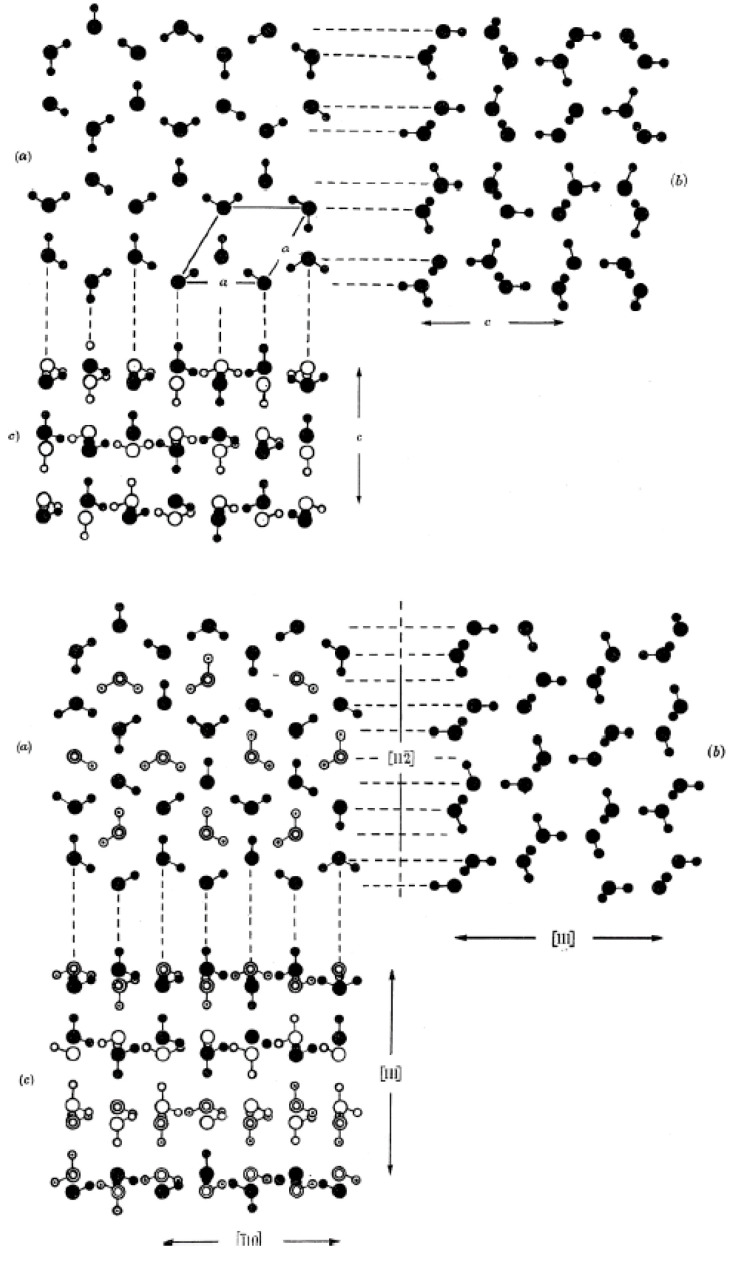
A schematic illustration of the crystal structures of hexagonal I_h_ (**top**) and cubic I_c_ (**bottom**). Image reproduced from [107], with permission from The Royal Society.

These results suggest that the critical nucleus size, and the concomitant nucleation work, may be substantially lower for the cubic *I*_c_ than for the hexagonal *I*_h_. The two ice structures have different crystallographic facets and each facet of both phases has its unique set of specific interfacial free energy values. The substantial difference in the nucleation work, notwithstanding the similarities between the crystal structures of hexagonal *I*_h_ and the cubic *I*_c_ [107,108] (Figure 5), suggest that the specific interfacial free energy value of each facet may be very sensitive to a minute change in the crystallographic structure of ice. 

We may note at this stage however that some of what has been thought to be *I*_c_ under a certain temperature range in the past could in fact have been what is called “stacking-disordered” ice which contains mixture of *I*_c_ layers and *I*_h_ layers [109]. A recent simulation study found that the entropy of mixing of these layers rendered such stacking-disordered ice more stable than either pure *I*_c_ or pure *I*_h_ [110]. 

## 7. Size Distributions of Ice Particles in the Atmosphere

A relevant matter that might have bearing on ice nucleation is the observed size distributions of ice particles in various parts of the atmosphere around the glove. This is a practically important issue as our ability to predict the size distributions of ice particles in the atmosphere is directly relevant to computation of number concentrations of ice in a climate model. Size distributions of ice particles in various parts of the atmosphere consistently show a trend that more smaller ice particles exist than larger ones [111]. This is puzzling because the Laplace pressure and hence the chemical potential of H_2_O molecule is higher in a smaller ice particle than in a larger one. Thermodynamically, the large ice particles are expected to grow at the expense of the smaller ones, and with time, size distributions are expected to be progressively more populated with larger ice particles. The opposite trend observed thus implies either (1) small particles are continually being generated due to a secondary nucleation that effectively multiplies the original nucleation events, (2) small particles are somehow prevented from growing even though it would be thermodynamically favorable for them to do so, (3) gravity removes the larger particles more effectively than anticipated or (4) existing ice particles are continually breaking up notwithstanding the higher free energy costs of doing so. The last scenario can be further subdivided into several possible mechanisms of break up [112]. No plausible explanation yet exists that can account for the observed size distribution of ice particles in the atmosphere [30,112]. Since only one of the many possible scenarios listed above could involve nucleation, it is possible that the secondary ice production has nothing to do with nucleation. Still, given the importance of the issue, we may briefly note the issue of secondary nucleation. 

The term secondary nucleation refers to a situation in which the presence of a nucleation site is not independent of the primary nucleation event. After an ice particle nucleates and starts to grow in a certain site in the bulk of continuous liquid water, invisibly tiny fragments of growing ice particles may break off and diffuse through the continuous liquid water phase. They may then act as a new seed of ice formation some distance away from the existing growing ice crystal. However, secondary nucleation is more difficult to envision in a continuous vapor phase. Unlike in a continuous liquid phase, the perceived new seed of ice would not be surrounded by an ample supply of water molecules in a vapor phase whose supersaturation must have fallen due to the primary nucleation and the subsequent growth of the primary nucleus. 

The atmosphere is an enormously complex system. It contains a myriad of particles and the leading candidates for heterogeneous nucleators in the atmosphere are mineral dust and emissions from aircraft like soot. Two common atmospheric clay particles are kaolinite and montmorillonite [30]. Glacial outwash, after the recent and geographically widespread retreat of glaciers, is a major source of mineral dust that has a remarkably high heterogeneous nucleation potency of ice in the atmosphere [113]. The atmosphere also contains salts, which are hygroscopic, and the pH of rainwater is not neutral but weakly acidic. Salts are highly soluble in water, so salts absorb and attract water. Consequently, airborne salts may grow to large droplets of salt solutions. The activity of water is lower in a salt solution than in pure water due to the entropy of mixing, so deeper supercoolings are required for the water in such droplets of salt solutions to freeze. Given all these variables and complexities, it may be some time before we know the answer. 

## 8. The Memory Effect in Ice Nucleation

One of the most important questions in heterogeneous nucleation of ice is; “what are the most important properties or functionalities of an effective ice nucleator?” The nucleation of ice has a long-standing mystery called “the memory effect” that likely has direct implications to the question. The term refers to a phenomenon whereby the surface of a heterogeneous nucleator of ice becomes somehow “conditioned” by nucleation of ice for the first time and renders the nucleation of ice easier for the second time [73,114,115,116]. The phenomenon may also be referred to as “pre-activation” or “evaporation nucleation”, and has been reported to occur after a solid nucleator has induced ice nucleation once or the nucleator has been cooled to below 233 K [117]. Importantly, it remains unclear exactly which property of the solid surface can be conditioned by the nucleation of ice for the first time. That the surface history of a solid particle makes a measurable difference in the nucleation potency of a heterogeneous nucleator might provide hints as to the search for the most important properties or functionalities of an effective ice nucleator. 

Early reports by Evans suggested two possibilities as to the cause of the memory effect in ice nucleation; either a trace of leftover ice layer on the nucleator surface or in the form of a structure imprinted on the nucleator surface by previous contact with ice [73]. If the memory were due to a structure imprinted on the nucleator surface, then the loss of the memory would be due to the relaxation and diffusion of atoms/molecules on the solid nucleator which is expected to occur near the Tammann temperature of the solid nucleator. It was reported that the memory was lost after moderate heating of the nucleator, to between 272 K and 274 K, and totally lost by heating to a temperature higher than 274 K, which was close to the melting of ice and far below the Tammann temperature of any solid nucleator tested. In addition, a control experiment using D_2_O in place of H_2_O (the melting point D_2_O of is 3.8 K higher than that of H_2_O [118]) showed that the memory was lost when the nucleator was heated to between 275 K and 277 K [73]. This threshold temperature of D_2_O above which the memory was lost was also 3 to 4 K higher than that of H_2_O. These findings suggest that the memory effect is the property of water, not the property of the solid surface of the nucleator that has been in contact with water. The initial explanations thus invoked some kind of leftover ice that might persist in small cracks, crevices, cavities or other surface defects above the melting point [73]. Later, it was found that the memory effect was due to an ordered, ice-like layer of water molecules on the solid surface which remained stable to temperatures well above the melting point [115,116]. 

In the early 1990s, Rosinski [119] and Rosinski and Morgan [120] reported another type of surface conditioning that led to the memory effect. In their study, particles were first exposed to supersaturated water vapor at a temperature below 273 K, which induced condensation of liquid water, not ice, and then the resulting droplets were evaporated. Next, the same particles were exposed to water vapor that was supersaturated with respect to ice but undersaturated with respect to liquid water at variable temperatures down to 253 K. Ice has a lower vapor pressure than liquid water at the same *P*/*T* condition, so it is possible to set a *P* that is supersaturated with respect to ice but undersaturated with respect to liquid water. Then, some of the treated particles formed ice crystals by deposition from the vapor phase. In a control experiment, none of the particles formed ice from the vapor phase without such conditioning. That a fraction of particles directly nucleated ice from vapor suggests imprinting of the memory by surface conditioning. The memory thus imprinted was sometimes deactivated after the ice was sublimed away and in other times persisted through several condensation-freezing-evaporation cycles. 

In the early 2000s, Seeley and Seidler investigated the effect of Langmuir films of alcohols of intermediate chain lengths (21, 23, or 25 carbon atoms per molecules) on the surface of water droplets on ice nucleation [121]. The water droplets sit on a borosilicate glass that had been hydrophobized with hexamethyldisilizane to render the receding contact angle of water to be around 90°. They let a chloroform solution that contained the alcohol spread on the surface of a 10 µl water droplet sample and let the chloroform evaporate, so that if distributed uniformly on the surface, the alcohol content would cover on average 1.5 monolayers. The lattice matching between the 2D structure of these Langmuir films and that of *I*_h_ perpendicular to the c-axis is close [71]. They found that the effectiveness of the alcohol film to ice nucleation was a strong function of the chain length of the alcohols in the film. The nucleation potency of the alcohol film was lost after the system was heated to a certain threshold temperature, like the memory effect in the earlier studies. Importantly, the effect of the surface conditioning was present when the system was cooled for the first time after the preparation of the Langmuir film (with no history of ice formation), as long as the Langmuir film was prepared below said threshold temperature [121]. Therefore, the term “memory effect” may not be suitable to describe the surface conditioning of ice nucleator because an alcohol film without any history of ice nucleation or contact with ice can still activate the effect. 

We point out that the findings of Seeley and Seidler do *not* contradict the earlier conclusion of Evans that the memory effect is due to a property of water as opposed to that of the surface of an ice nucleator. Given the practical difficulty of isolating all stages of sample preparation from the ambient moisture in addition to the practical unfeasibility of totally drying an alcohol, minute amount of water must have already been present on a freshly prepared Langmuir film of a hydrophilic alcohol monolayer. 

## 9. Surface Nucleation and Contact Nucleation

**Figure 6:**
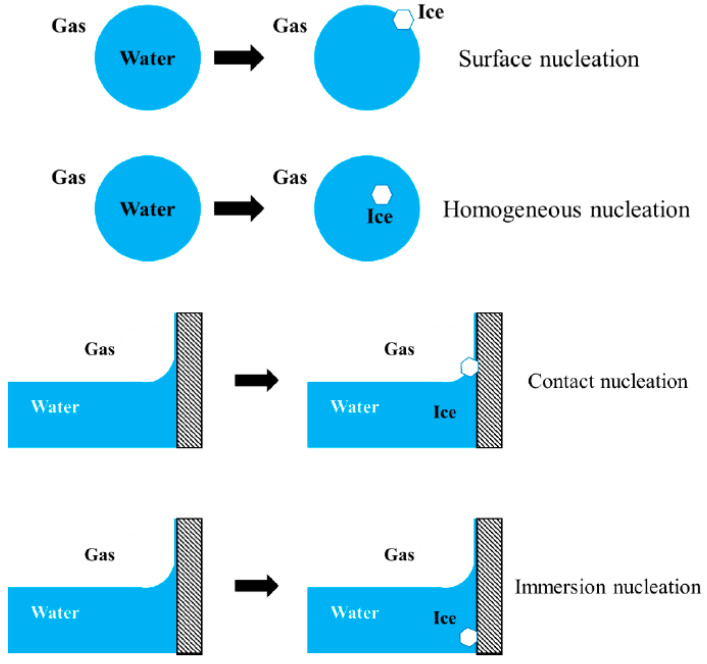
A schematic illustration of surface nucleation vs. homogeneous nucleation in the absence of a foreign solid wall (**top**) and contact nucleation vs. immersion nucleation in the presence of a foreign solid wall (**bottom**).

The term “surface nucleation” refers to the higher nucleation rate of ice at the surface of liquid water (e.g., the surface of a freely floating water droplet) than that of ice in the interior of the liquid water (homogeneous nucleation). The term “contact nucleation” refers to the higher nucleation rate of ice at the surface of liquid water in the presence of a foreign solid wall (e.g., around a three-phase line or a meniscus) than that of ice on the foreign solid wall that is immersed deep inside the liquid water (“immersion nucleation”) [122,123,124,125,126,127,128,129,130,131,132]. These configurations are schematically depicted in Figure 6. 

Contact nucleation (that involves a foreign solid) is thus a special case of surface nucleation [133]. In either case, the nucleation rate of ice next to a (liquid water)–(water vapor) interface is higher than that is away from the (liquid water)–(water vapor) interface. Thus, unlike the memory effect discussed above that concerned with a solid–water interface, surface nucleation and contact nucleation concern with a (liquid water)–(water vapor) interface. 

Surface nucleation suggests that the nucleation rate of ice under a given temperature –pressure condition will increase when a given mass of liquid water is sub-divided into many small masses, because the total surface area will increase substantially by such sub-division. It follows that the probability of observing formation of ice in at least a part of such sub-divided masses (e.g., mists for surface nucleation and aerosols for contact nucleation) would be higher than that in a large mass of liquid water. 

Intuitively, the concepts of surface nucleation and contact nucleation make sense. The system symmetry breaks at a (liquid water)–(water vapor) interface, so nucleation at a (liquid water)–(water vapor) interface is less isotropic or less “homogeneous” compared to nucleation in the interior of a single phase. Since homogeneous nucleation is supposed to require a greater activation barrier to be surmounted than heterogeneous nucleation, a greater level of system symmetry is expected to result in a more homogeneous system and hence a lower nucleation rate. Though conceptually sensible, a precise mechanism of surface nucleation or contact nucleation remains unclear [134]. 

Ubiquitous presence of pits, defects and other irregularities on the surface of a solid particle may suggest that a situation schematically depicted in Figure 4 may be common. The mouth of the narrow wedge, before the ice has nucleated and grown as depicted in Figure 4, would be nothing other than a typical configuration of contact nucleation. Indeed, contact nucleation has been found to be of central importance in heterogeneous nucleation of ice. For example, the nucleation potency of AgI has been found to be substantially more effective in the contact mode than in the immersion mode [122]. In contrast, Duft and Leisner reported that surface nucleation may be unimportant in homogeneous nucleation of water droplets, only potentially important for water droplets that are much smaller than 20 µm, if at all [135]. 

It has been found that many heterogeneous nucleators have different nucleation thresholds when they act in different modes of contact or immersion [127]. These results suggest that a given solid nucleator may act in different ways depending on whether it is totally immersed in liquid water or partially extrudes to the vapor phase. The cause of surface nucleation/contact nucleation is unknown but it is suggested that (liquid water)–(water vapor) interface is of special interest in ice nucleation [127]. 

Such enhancement of the nucleation of a solid phase by its liquid–vapor interface occurs in other systems. The most extreme examples of such enhancement by a gaseous phase may be surface freezing of normal alkanes of intermediate chain lengths [136] and alcohols [137]. In either case, a monolayer of normal alkane or alcohol forms at the liquid melt–vapor interface slightly above the melting point of the respective alkane [136] or alcohol [137]. Formation of such a monolayer of the solid phase is itself an activation process (two–dimensional nucleation) due to the line tension at the periphery of a flat disk of the new phase [138]. Surface freezing occurs on a free liquid melt–vapor interface [136] as well as on an isolated single solid substrate [139,140], but not when sandwiched between two such solid substrates (when not exposed to the vapor) [140]. These findings show that a gaseous phase, or a vapor phase, is far from “nothing” and its presence cannot be ignored in the nucleation of a solid phase. 

We now return to the surface nucleation/contact nucleation of ice. Using computer simulation, Sear calculated the nucleation rates of ice at the three-phase-line (where the liquid water, the solid substrate and the water vapor meet), at interfaces and in the bulk liquid water. He found that the nucleation rate at the three-phase-line is orders of magnitude higher than it is anywhere else [129]. Classical nucleation theory calculations suggest that this finding should be generic [127,141]. Surface nucleation becomes thermodynamically favorable to bulk nucleation when the condition of partial wetting of at least one of the facets of the crystal nucleus by its own melt is satisfied [127]. Such partial wetting of a solid surface by its own melt was experimentally observed for several systems [142,143], including water-ice at temperatures at or below 273 K [144], and is consistent with the recent finding that the pre-melting layer of ice is of partial wetting nature (in the form of patches rather than a continuous two-dimensional layer as the term “pre-melting layer” may suggest) [145]. 

What we have discussed so far in this section is based on an assumption that the system is clean and free of any organic contaminants. We saw in the previous sections that some organic compounds have surprisingly high ice nucleation potency. Since these organic compounds generally have lower specific surface energy values than that of water or ice, they are likely present at the surface of water than in the interior of bulk water. Thus, care must be taken to ensure that the system is free of such organic compounds, or nucleation of ice due to (unintended or undetected) presence of such ice nucleator at the surface of liquid water could potentially be mistakenly interpreted as surface nucleation. 

## 10. Concluding Remarks

After a brief introduction of key general concepts like classical nucleation theory and the Wulff theorem, we have focused on several selected unresolved issues in ice nucleation, such as the unexpectedly poor heterogeneous nucleation potency of lattice-matching nucleators, unexpectedly good heterogeneous nucleation potency of organic substances that have no structural similarities to ice, possible multi-step mechanisms of nucleation of ice, system size dependence, potential secondary nucleation in the atmosphere, the memory effect, surface nucleation, and contact nucleation. Each of these issues remains unresolved and its elucidation is expected to shed new and important light to our understanding of ice nucleation. It appears that the ordering of the water molecules next to an interface is the key determining factor of ice nucleation potency of a heterogeneous ice nucleator. 

It emerged that the problems are two-fold. On the one hand, a molecular-level understanding of ice nucleation at an interface will be required to elucidate the issue of the unexpectedly poor heterogeneous nucleation potency of lattice-matching nucleators, the unexpectedly good heterogeneous nucleation potency of organic substances and the memory effect. Such understanding would also aid design strategies of effective heterogeneous nucleation promotors of ice. On the other hand, a new and comprehensive theory will be required to surmount the limitations in classical nucleation theory that most acutely show in heterogeneous nucleation of ice from water vapor. 

## Nomenclature

*J*	nucleation rate
*A*	a kinetic constant that includes the attachment frequency of monomer molecules
*N*_0_	the concentration of potential nucleation sites
Δ*g*	the height of the activation barrier
*k*	the Boltzmann constant
*T*	the absolute temperature
*g*(*r*)_bulk_	the free energy of forming a new phase at a constant temperature in the absence of an interface
*g*(*r*)_interfacial_	the interfacial free energy between the new phase and the old phase at a constant temperature
Δ*g*(*r*)_activation_	the height of the activation barrier at a constant temperature, which is the sum of *g*(*r*)_bulk_ and *g*(*r*)_interfacial_
*r*	the radius of the nucleus
*P*	the actual vapor pressure
*P*_0_	the saturation vapor pressure
γ	the specific interfacial free energy
*v*_m_	the molecular volume
*r*_K_	the Kelvin radius. For a spherical nucleus, *r* = 2*r*_K_
*n*	number of molecules in a nucleus
*n**	number of molecules in a critically sized nucleus
θ	the contact angle of a spherical cap–shaped nucleus that forms on the substrate in the metastable parent phase
*W*(*n*)	nucleation work, which is a function of the size of the nucleus
